# Non-Cancer Chronic Pain Conditions and Risk for Incident Alzheimer’s Disease and Related Dementias in Community-Dwelling Older Adults: A Population-Based Retrospective Cohort Study of United States Medicare Beneficiaries, 2001–2013

**DOI:** 10.3390/ijerph17155454

**Published:** 2020-07-29

**Authors:** Sumaira Khalid, Usha Sambamoorthi, Kim E. Innes

**Affiliations:** 1 Department of Epidemiology, West Virginia University School of Public Health, Morgantown, WV 26506, USA; karen.innes@hsc.wvu.edu; 2 Department of Pharmaceutical Systems and Policy, West Virginia University School of Pharmacy, Morgantown, WV 26506, USA; usambamoorthi@hsc.wvu.edu

**Keywords:** Alzheimer’s disease, dementia, ADRD, modifiable risk factors, Chronic pain, elderly population, longitudinal study, arthritis, headache

## Abstract

Accumulating evidence suggests that certain chronic pain conditions may increase risk for incident Alzheimer’s disease and related dementias (ADRD). Rigorous longitudinal research remains relatively sparse, and the relation of overall chronic pain condition burden to ADRD risk remains little studied, as has the potential mediating role of sleep and mood disorders. In this retrospective cohort study, we investigated the association of common non-cancer chronic pain conditions (NCPC) at baseline to subsequent risk for incident ADRD, and assessed the potential mediating effects of mood and sleep disorders, using baseline and 2-year follow-up data using 11 pooled cohorts (2001–2013) drawn from the U.S. Medicare Current Beneficiaries Survey (MCBS). The study sample comprised 16,934 community-dwelling adults aged ≥65 and ADRD-free at baseline. NCPC included: headache, osteoarthritis, joint pain, back or neck pain, and neuropathic pain, ascertained using claims data; incident ADRD (N = 1149) was identified using claims and survey data. NCPC at baseline remained associated with incident ADRD after adjustment for sociodemographics, lifestyle characteristics, medical history, medications, and other factors (adjusted odds ratio (AOR) for any vs. no NCPC = 1.21, 95% confidence interval (CI) = 1.04–1.40; *p* = 0.003); the strength and magnitude of this association rose significantly with increasing number of diagnosed NCPCs (AOR for 4+ vs. 0 conditions = 1.91, CI = 1.31–2.80, *p*-trend < 0.00001). Inclusion of sleep disorders and/or depression/anxiety modestly reduced these risk estimates. Sensitivity analyses yielded similar findings. NCPC was significantly and positively associated with incident ADRD; this association may be partially mediated by mood and sleep disorders. Additional prospective studies with longer-term follow-up are warranted to confirm and extend our findings.

## 1. Introduction

Alzheimer’s disease and related dementia (ADRD) is a group of serious neurodegenerative disorders characterized by progressive decline in cognitive and psychomotor function [1,2]. Prevalence of ADRD continues to increase worldwide, exacting enormous social, economic and healthcare costs. For example, in the United States, at least 5.8 million adults are living with Alzheimer’s disease (AD), the most common form of dementia, with attributable Medicare costs alone exceeding $146 billion annually; these figures are expected to rise steeply in coming years [3]. ADRD is also a significant contributor to mortality and number of life years lived with disability [3,4]. With no cure for ADRD, and no effective disease-modifying treatments yet available despite decades of clinical research [5], efforts are increasingly shifting to prevention and early intervention, including intensive approaches to identify and target modifiable risk factors for ADRD [6].

To date, several factors have been linked to elevated ADRD risk. These include non-modifiable factors such as advancing age, female sex, rurality, and specific genetic profiles, as well as modifiable risk factors [7]. The latter include: poor education and poverty [7,8]; physical inactivity, midlife obesity, and other lifestyle factors [7,9]; history of head injury or stroke [10,11]; and certain chronic physical health disorders (e.g., diabetes, cardiovascular disease, midlife hypertension, respiratory illness [7,9,12]). Specific mental health conditions (e.g., depression, anxiety) and chronic sleep impairment have also been shown to predict subsequent cognitive decline and conversion to ADRD [13,14,15,16]. In addition, there is growing evidence from observational and experimental research that chronic pain, an increasingly common and highly burdensome condition, may also contribute to elevated risk for neurocognitive impairment and development of ADRD [17,18,19,20,21,22,23]. 

Although limited at present, there is evidence from longitudinal studies that chronic pain and common non-cancer chronic pain conditions (NCPC) may increase risk for incident cognitive impairment and ADRD [24,25,26,27,28,29,30,31,32,33,34]. For example, recent retrospective cohort investigations of Taiwanese nationals [26,28,29,34] and prospective cohort studies of Japanese and Norwegian elders [25,27,33] have reported significantly increased risk of incident dementia in adults previously diagnosed with fibromyalgia [28], osteoarthritis or knee pain [26,33], and headache [25,27,29,34]. Likewise, findings from a handful of longitudinal studies in US [24,30,32] and British adults [31] suggest that non-specific chronic pain may predict subsequent deterioration in memory [30,32], accelerated cognitive decline [32], new onset cognitive impairment [32], and incident dementia [24,32], although studies varied widely in design, study population, length of follow-up, and measures. However, to our knowledge, no study has investigated the collective and incremental association of common chronic pain conditions to ADRD risk, nor the potential mediating role of depression, anxiety and sleep disorders, conditions strongly and bidirectionally associated with chronic pain and linked to ADRD risk. 

In this study, we investigate the association of common NCPC to ADRD risk using multiple retrospective cohorts from a linked database of nationally representative sample of older US Medicare beneficiaries. We hypothesized that the presence of NCPC at baseline will be associated with significantly increased risk of incident ADRD, and that the magnitude of this association will increase with rising number of NCPC at baseline. We further hypothesized that depression, anxiety and/or sleep impairment, will partially mediate the association between NCPC and incident ADRD.

## 2. Methods

### 2.1. Study Design

Our study used a retrospective cohort design to assess the association of baseline common non-cancer chronic pain conditions (NCPC) to incident ADRD using data from the Medicare Current Beneficiary Survey (MCBS) [35] linked with Medicare fee-for-service claims. MCBS is a nationally representative survey of adults participating in Medicare health insurance program. As detailed below, multiple three-year MCBS cohorts (2001–2013) were pooled to construct the analytic sample for this study.

### 2.2. Data Source

First initiated in 1991, MCBS has been recruiting survey participants each year using a complex stratified, three-stage probability sampling design described in detail elsewhere [36,37]. MCBS participants undertake structured in-person interviews at baseline and follow-up rounds each year for up to three years. MCBS generates comprehensive cross-sectional and longitudinal data on participants’ health status, health services utilization, prescription medications, and payment sources using a combination of survey and administrative records. Our study used data from MCBS Cost and Use files linked with Medicare fee-for-service claims to ascertain demographics, access to care, lifestyle factors, medical history and medication. Based on the recommendations by MCBS investigators [36,37,38] and sampling strategies documented in prior published studies using MCBS [36,38,39], we combined 11 MCBS cohorts in order to maximize reliability and precision of our study estimates; these included the following cohorts: 2001–2003; 2002–2004; 2003–2005; 2004–2006; 2005–2007; 2006–2008; 2007–2009; 2008–2010; 2009–2011; 2010–2012 and 2011–2013.

### 2.3. Study Sample

The study sample comprised continuously fee-for-service enrolled community-dwelling Medicare beneficiaries, aged 65 years or over, who had complete information on NCPC status and were still alive at the end of follow-up. Institutionalized participants were excluded, as were participants with diagnosed ADRD at baseline. As depicted in the sample selection flow chart (see supplementary Figure S1), application of all a priori exclusion criteria yielded a final sample size of 16,934 adults (N’s by year = 1874 (2001–2003); 1768 (2002–2004); 1884 (2003–2005); 1766 (2004–2006); 1735 (2005–2007); 1650 (2006–2008); 1495 (2007–2009); 1278 (2008–2010); 1034 (2009–2011); 1159 (2010–2012); and 1291 (2011–2013)). 

IRB/ethics approval: The present study was approved as exempt protocol by the WVU Institutional Review Board (IRB), due to the deidentified nature of the data used in the study.

### 2.4. Dependent Variable: Incident Alzheimer’s Disease and Related Dementia (ADRD) at Follow-Up– Yes/No

The Medicare fee-for-service (FFS) claims for inpatient (IP), skilled nursing facility (SNF), outpatient (OT), home health agency (HHA), and physician office (PO) visits for years 2001–2013 as well as MCBS self-reported Health Status and Functioning files were used to ascertain the presence of ADRD at baseline and follow-up. The presence of ADRD at both baseline (year 1) and follow-up (years 2 and 3) was ascertained using a validated CMS algorithm (Centers for Medicare and Medicaid Services (CMS) Chronic Condition Algorithms) [40] of at least one fee-for-service claim with any of these International Classification of Diseases, ninth Edition, clinical modification (ICD-9-CM) diagnostic codes: 331.0, 331.11, 331.19, 331.2, 331.7, 290.0, 290.10, 290.11, 290.12, 290.13, 290.20, 290.21, 290.3, 290.40, 290.41, 290.42, 290.43, 294.0, 294.10, 294.11, 294.20, 294.21, 294.8, and 797, or an affirmative response to the self-reported Health Status question “Has a doctor ever told you that you had Alzheimer’s?” [41]. Using a combination of claims and survey data to ascertain ADRD has been recommended by MCBS investigators to increase capture of ADRD and has been shown to yield results similar to those of expert in-person-assessment [42].

### 2.5. Key Independent Variable: Non-Cancer Chronic Pain Condition (NCPC) and Number of NCPCs

Baseline NCPC were identified using Medicare fee-for-service claims. NCPC included five common NCPC (back or neck pain, headache, joint pain, neuropathic pain, and osteoarthritis). The presence of any NCPC was identified using either two outpatient claims (90 days apart) or one inpatient claim using ICD9-CM codes as recommended by the Centers for Medicare and Medicaid Services [40] and consistent with prior studies of NCPC [43,44]. Any NCPC was assessed as a binary variable (yes/no) during baseline. Relative NCPC burden was ascertained with a count variable (0–5 NCPCs). 

### 2.6. Covariates

To account for the influence of potential confounding, specific baseline characteristics known or suspected to be associated with ADRD risk and/or chronic pain based on prior research were selected a priori for inclusion as covariates in our multivariable models. These included age group (65–69, 70–74, 75–79, and ≥80 years), based on prior research indicating the risk in adults doubles approximately every 4 years after age 65 [3,45]. Other biological factors i.e., sex (female/male), and race/ethnicity (Non-Hispanic White, African American, Hispanic, and other); other demographic characteristics, i.e., marital status (married, widowed, divorce/separated, other), educational level (less than high school, high school, some college, college), family income, measured as percentage of federal poverty line (FPL) (poor/low-income (<200%), (middle to high-income (≥200%)); health insurance status (insured by Medicaid (yes/no) or private insurance (yes/no)); rurality (metropolitan areas, yes/no); and lifestyle factors, i.e., smoking status (current smoker, past smoker, never smoked) and body mass index (BMI, kg/m^2^) (underweight (<18.5), normal (18.5–24.9), overweight (25–29.9), and obese ≥30). 

Also included as covariates were other chronic physical health conditions reported at baseline (yes/no). These included hypertension, diabetes, heart disease, respiratory illness, and history of stroke, traumatic brain injury (TBI), and cancer (with the exception of non-melanotic skin cancer), as well as specific auto-immune conditions associated with chronic pain, including rheumatoid arthritis (RA), and systemic lupus erythematosus (SLE). In addition, certain commonly used medications that have been linked to ADRD risk and/or used in chronic pain management were also evaluated as covariates. These included non-steroidal anti-inflammatory drugs (NSAIDS) [46], opioid analgesics [47], benzodiazepines [48] and certain other psychotropic drugs (e.g., selective serotonin reuptake inhibitors (SSRIs), serotonin norepinephrine reuptake inhibitors (SNRIs), monoamino oxidase inhibitors (MAOIs), tricyclic antidepressants (TCAs), and atypical antidepressants) [49]. Benzodiazepine use was evaluated as a separate medication category given that: (1) a significant number of study participants (10%) were prescribed these medications; and (2) prior research has suggested that benzodiazepines not only has multidimensional neurological effects [50,51], but is also associated with elevated risk for ADRD [48].

*Mediators:* To examine the potential mediating influence of mood and sleep disorders on the relation of NCPC to incident ADRD, we evaluated the effects of diagnosed depression, anxiety and insomnia-related sleep disorders both separately and in combination (see supplementary Table S1 for ICD-9-CM diagnostic codes). 

### 2.7. Statistical Analysis

Potential differences in baseline characteristics by NCPC and ADRD status were determined using Rao-Scott chi-square tests (categorical variables). Logistic regressions were used to assess the unadjusted and independent association of NCPC and NCPC burden to incident ADRD. In these multivariable logistic regressions, we carried out block-wise adjustment for demographic and socioeconomic factors; lifestyle factors and chronic physical health conditions; and analgesics and psychotropic medications to assess the adjusted associations of NCPC to ADRD risk. Similar nested multivariable logistic regression models were used to evaluate potential mediating effects of baseline diagnoses of depression, anxiety, and sleep disorders both individually and in combination. To assess the linear relationship of NCPC to risk of ADRD, regression models with polynomial contrast were used. For our sensitivity analyses, we used multinomial logistic regression to build competing risk of death models with the following four outcomes: ADRD-free and alive at the end of follow-up (N = 15,785) (referent category); ADRD-free and not alive at the end of follow-up (N = 1220); ADRD positive and Alive at the end of follow-up (N = 1149); ADRD positive and not alive at the end of follow-up (N = 259). 

All bivariate and multivariable analyses were carried out using MCBS complex survey design sampling weights. MCBS data-cycles are released with cross-sectional as well as longitudinal weights. We used 3-year backward longitudinal weights for analysis of our pooled cohorts to yield up to two years of continuous follow-up. All analyses were performed using SAS survey procedure (SAS version 9.4, SAS Institute, Inc.).

## 3. Results

Our analytical sample comprised 16,934 eligible participants with a mean age at baseline of 74 ± 0.07 years. The majority of the cohort were female (57%), white (83%), married (56.5%) and reported a family income at or above 200% of the FPL. Study participants were predominantly from metropolitan areas (71%) and most had private insurance (79%).

Overall baseline prevalence of any NCPC in this study was 36% (weighted), with significant differences by cohort (range 31% to 39%, *p* < 0.0001). Pain burden in this population was high, with 37.5% reporting at least two co-existing NCPCs and 15% indicating three or more NCPCs. A significantly higher percentage of participants with baseline NCPC reported a history of chronic physical health conditions associated with ADRD, including hypertension (82% vs. 68.7%), heart disease (31.5% vs. 19.9%), stroke (14% vs. 10%), diabetes (23.5% vs. 19%) (*p*’s < 0.0001). Participants with baseline NCPC were also significantly more likely to report mood disorders, including depression (11% vs. 4.5%) and anxiety (7.9% vs. 3.5%), as well as sleep disorders (25.5% vs. 10.2%) when compared to participants without baseline NCPC (Table 1).

Table 2 summarizes the baseline characteristics of the study sample by incident ADRD status. A total of 1149 participants were diagnosed with incident ADRD during the 2-year follow-up period (overall incidence rate = 6.8 per 100 participants). Incident ADRD rates did not differ by study cohorts (range 4.9 to 6.2 per 100, *p* = 0.9), and were significantly higher in women (6.2% vs. 5.0% in men), and in participants who were black (7.4% vs. 5.5% in Non-Hispanic whites), widowed (8.2% vs. 4.5% in married), and more poorly educated (*p*’s < 0.0001). The proportion of participants diagnosed with incident ADRD was also significantly higher in those who were <200% of the federal poverty level (7.4% vs. 4.3%), were on Medicaid (9.1% vs. 5.2%) and who lacked private insurance (7.2% vs. 5.3%) (*p*’s < 0.0001). In addition, the percentage diagnosed with ADRD was significantly greater in those who indicated a history of specific physical health conditions at baseline, including hypertension, heart disease, stroke, and TBI, as well as in those who reported use of opioid analgesics (6.9% vs. 5.4%), benzodiazepines (8.5% vs. 5.4%), or other psychotropics (9.8% vs. 4.8%) (*p*’s < 0.0001). The proportion of participants with incident ADRD increased significantly with rising number of comorbid physical health conditions (range 4.6% to 10.6%, *p* < 0.0001). Participants who reported sleep, depression or anxiety disorders at baseline also included a significantly higher proportion of ADRD cases (*p*’s < 0.0001). Notably, as detailed in Table 2, incident ADRD rates were significantly higher in those who reported any NCPC at baseline (6.6 vs. 4.3 per 100), and rose with increasing number of NCPC conditions, from 6.2 in those with 1 NCPC condition to 17.3 per 100 participants in those with 4 or more NCPC conditions (*p*’s < 0.0001).

### 3.1. Association of NCPC to Incident ADRD

In our unadjusted logistic regression model, those with baseline NCPC were 54% more likely to have incident ADRD (odds ratio (OR) = 1.54, 95% confidence interval (CI) = 1.34–1.78, *p* < 0.0001) (Table 3). Adjustment for demographic and socioeconomic characteristics (sex, race, age, education, poverty, Medicaid insurance, private insurance, and marital status) attenuated but did not eliminate this association (adjusted OR (AOR) = 1.33, 95% confidence interval (CI) =1.14–1.53, *p* < 0.0001). 

The magnitude of this association was modestly reduced after additional adjustment for lifestyle factors and comorbid physical health conditions (AOR = 1.28, CI 1.10–1.48, *p* = 0.001), and further attenuated by inclusion of medications in the model (AOR adjusted for NSAIDs and opioid analgesics (AOR = 1.25, CI 1.08–1.45, *p* = 0.003) (Table 3). Further adjustment for benzodiazepines and other psychotropics only slightly reduced the magnitude of this estimate (AOR = 1.21 (1.04–1.40, *p* = 0.01)). As detailed in Table 3, the association of NCPC to incident ADRD rose significantly in both strength and magnitude with increasing number of pain conditions. Relative to beneficiaries with no NCPC at baseline, those with ≥4 NCPCs were twice as likely to be subsequently diagnosed with ADRD after adjustment for demographics, socioeconomic status, lifestyle factors, and medical conditions (AOR = 1.98, CI = 1.36–2.89, *p*-trend <0.00001). Further adjustment for analgesic medications only slightly attenuated these associations (AOR for ≥4 vs. no NCPC = 1.91, CI 1.31–2.80, *p*-trend = 0.0008). When number of NCPCs at baseline were assessed as a continuous variable, risk for incident ADRD increased 12% for each additional NCPC in the fully adjusted model (AOR = 1.12, CI = 1.06–1.20, *p* = 0.0007).

### 3.2. Sensitivity Analyses

Analyses using competing risk of death models yielded findings consistent with those from our primary analyses (Table 4). Relative to participants without NCPC at baseline, those with any NCPC and still alive at follow-up were significantly more likely to be diagnosed with incident ADRD after adjustment for demographics, lifestyle characteristics, physical health conditions, analgesics, and other factors (AOR for any NCPC = 1.26, 95% CI = 1.08–1.46, *p* < 0.003). In contrast, baseline NCPC status was unrelated to death during the follow-up period, either with or without a diagnosis of incident ADRD (AORs, respectively = 0.99 and 0.97, *p*’s ≥ 0.6). Further adjustment for psychotropics did not appreciably change these estimates. These findings suggest survival bias is unlikely to explain the positive association observed between baseline NCPC and incident ADRD.

### 3.3. Potential Mediating Effects of Sleep and Mood Disorders

As illustrated in Table 5, inclusion of mood disorders in fully adjusted model modestly attenuated the relation of NCPC to incident ADRD (AORs for any NCPC and 4+ NCPCs vs. no NCPC, respectively = 1.17 (1.01–1.37) and 1.57 (1.06–2.34), *p*’s ≤ 0.04). Likewise, the addition of sleep disorders to the model attenuated the association of NCPC to ADRD risk (AORs for any NCPC and 4+ NCPCs vs. no NCPC, respectively = 1.20 (1.04–1.39) and 1.66 (1.12–2.45) *p*’s < 0.02) as did that of both sleep and mood disorders (AORs for any NCPC and 4+ NCPCs, respectively = 1.18 (1.01–1.38) and 1.41 (0.94–2.12)). Adding mood and sleep disorders to models adjusted only for sociodemographics yielded similar risk estimates (AORs for any NCPC = 1.19, CI 1.02–1.47, *p* < 0.05), again suggesting that mood and sleep disorders may in part mediate the relation of NCPC to incident ADRD. Additional adjustment for benzodiazepines and psychotropics did not appreciably alter these associations (AOR = 1.17, CI = 1.01–1.37, *p* = 0.04). A number of demographic, lifestyle, and health-related factors remained significantly associated with incident ADRD in our fully adjusted models. These include: black (vs. non-Hispanic white) race (AOR = 1.48, CI 1.09–2.00); age (80+ vs. 65–69, AOR 6.12, CI = 4.7–7.9); poverty (<200% vs. >200%, AOR 1.17, CI = 1.02–1.34); Medicaid insurance (AOR 1.27, 95% CI = 1.01–1.6); BMI (underweight vs. normal, AOR = 1.48, CI = 1.04–2.12); history of stroke (AOR 1.87, CI = 1.6–2.2) or TBI (AOR = 1.52, CI = 1.04–2.23); and psychotropic medications (AOR = 2.04, CI = 1.74–2.39) (*p*’s < 0.05). Use of neither NSAIDs nor opioids was significantly associated with ADRD risk in the adjusted analyses (AORs respectively = 0.96 (0.80–1.16) and 1.03 (0.87–1.22)), nor was use of benzodiazepines (AOR = 1.17, CI = 0.97–1.40) (*p*’s > 0.05).

## 4. Discussion

In this retrospective cohort study of older community-dwelling Medicare fee-for-service enrollees, diagnosed NCPC at baseline remained significantly and positively associated with risk for incident ADRD after adjustment for demographics, socioeconomic factors, medical history, medications, and other factors. The strength and magnitude of this association rose with increasing number of NCPCs, indicating increasing risk for incident ADRD with rising chronic pain condition burden. The relation between NCPC and risk of ADRD appeared to be mediated in part by the presence of sleep and mood disorders. To our knowledge, this investigation is the first large retrospective cohort study to assess the collective and incremental association of NCPC to incident ADRD, and to explore the potential mediating role of mood and sleep disorders in this association. 

The significant positive associations NCPC to ADRD risk observed in this study are consistent with those of prior longitudinal studies regarding the relation of specific chronic pain conditions to risk for dementia. Recent large retrospective matched cohort studies in Taiwanese nationals [26,28,29,34,52], prospective cohort investigations in Norwegian adults [25,27] and a small retrospective cohort study of Canadian elders [53] all indicated significantly increased risk for incident all-cause dementia in those diagnosed with headache [25,27,29,34,52,53], OA/knee pain [26,33], and fibromyalgia [28] after adjusting for demographics, comorbid conditions, and other potential confounders. Although the few longitudinal studies investigating the association of non-specific chronic pain to subsequent cognitive deterioration or incident ADRD, all in British [31] and U.S. adults [24,30,32], have varied widely in study population, design, and methodology, results have likewise indicated that severe chronic pain [30,31], persistent pain [32], and/or reported pain interference [24] may predict subsequent worsening in memory [30,31], accelerated cognitive decline [32], and dementia [24,32].

While some longitudinal studies investigating the association between pain and dementia risk have included depression and/or anxiety as covariates in their adjusted models [24,28,29,30,32,34], no studies to our knowledge have explored the potential mediating role of mood disorders or evaluated the influence of sleep impairment on the association of chronic pain to incident ADRD. Sleep and mood disorders are common in older adults, have been strongly and reciprocally associated with chronic pain [54,55], and have been shown to be independent risk factors for ADRD [13,14,15,56,57], suggesting that sleep and mood impairment may mediate the observed associations between pain and ADRD risk. The role of sleep and mood neuromodulators, such as serotonin [58,59], dopamine and histamine [60,61] has been well documented in pain expression, likely contributing to the documented bidirectional relationships of sleep [54] and mood disorders [55,62] to pain conditions. In the current study, inclusion of depression, anxiety, and insomnia-related sleep disorders in the model weakened but did not eliminate the observed associations between NCPC and incident ADRD risk, suggesting a partial mediating role. In agreement with prior research, sleep [15,54] and mood disorders [13,14,57] remained strongly and positively associated with both baseline NCPC and with risk for incident ADRD in this cohort after adjustment for other factors.

*NCPC and increased ADRD risk; possible pathways:* although the mechanisms underlying the observed association of NCPC to incident ADRD remain speculative, chronic pain may operate via several pathways to increase risk for dementia [21,23,63,64,65]. Adults experiencing chronic pain have demonstrated diminished attention, impaired learning and memory, altered processing speed, reduced psychomotor efficiency, and compromised executive function [17,18,19,22,23], hallmarks of cognitive decline that may ultimately presage the development of cognitive impairment and dementia. Contributing to the documented decline in cognitive function that accompanies chronic pain, NCPC may promote specific adverse neurostructural and neurofunctional changes. For example, experimental and neuroimaging studies have demonstrated neurodegenerative changes in subjects with chronic pain that parallel those observed in ADRD [21,65,66], including reduction in grey matter volume in the amygdala, hippocampus and frontal cortices, the brain regions integrally involved in cognitive and behavioral functioning [66,67]. The increases in both peripheral and systemic inflammation that have been linked to chronic pain [68,69] may contribute to these neurodegenerative changes by contributing to neuroinflammation [63]. For example, chronic pain-induced microglial neuroinflammation has been directly implicated in Alzheimer’s disease pathogenesis via production of amyloid beta plaques and neurofibrillary tangles [21]; persistent inflammation negatively affects neuroplasticity and synaptic performance via reduction in brain-derived neurotrophic factors [66,68,69]. Neuronal receptors in the brain are neither infinite nor mutually exclusive and serve a range of neurologic functions under limited resources. During persistent pain, nerve endings fire rapid pain impulses to the brain for remedial actions, which in turn, exhausts the neuronal resources that are also involved in cognitive functions [69,70,71]. In addition, the presence of chronic pain conditions has been correlated with dysregulation of noradrenergic-modulated endogenous pain autoinhibition [69], which has, in turn, been linked to negative cognitive outcomes, including decline in working and long-term memory [70,71]. 

In agreement with previously published research [3,7,45], risk for ADRD increased strongly with age in this study, and was elevated among African Americans and in adults who had less education or lower family income, were on Medicaid, or had a history of diabetes, stroke, or TBI. Similarly, the significant positive relationships between baseline sleep and mood disorders and likelihood of incident ADRD observed in this sample of US Medicare beneficiaries are consistent with the findings of numerous prior investigations [14,15]. Both psychotropics and analgesics are frequently employed to manage chronic mood disorders, insomnia, and pain conditions in the older population. NSAIDs have been reported to reduce risk of ADRD in most but not all studies [46], whereas prior research has demonstrated modest or no association of opioid analgesics to ADRD risk [47]. In this study, use of neither NSAIDs nor opioid analgesics was significantly related to risk for ADRD. In agreement with some [49] but not all previous studies [48,72], use of psychotropics, but not benzodiazepines, remained significantly associated with incident ADRD after adjustment for multiple confounders. However, as noted above, adjustment for these medications only slightly attenuated the observed relationships of NCPCs to incident ADRD.

### Strengths and Limitations

This study has several strengths, including the population-based design, the use of longitudinal data from multiple cohorts, and the large, nationally representative sample of U.S. community-dwelling elders enrolled in FFS Medicare plans. Comprehensive information was available on a broad range of demographic and lifestyle characteristics, as well as on medical history, medication use, and other factors, allowing us to assess the potential confounding influence of these factors. Furthermore, NCPC’s, medication use, and history of other health conditions were ascertained using claims data and established algorithms. ADRD was identified using a combination of Medicare claims and survey data, likely leading to greater capture of this often under-reported and underdiagnosed outcome [41,73,74]. Notably, previous studies have indicated high specificity (89-95%) and acceptable sensitivity (64-85%) for the ascertainment of ADRD using multiple years of Medicare claims data [41,73,74]. We used a 3-year backward cohort design, with a two-year continuous follow-up and incident ADRD measured at two time points. 

Our study also has a number of limitations, including the relatively short follow-up period and lack of information on NCPC duration or on chronic pain symptoms, precluding a more comprehensive assessment of the potential role of chronic pain and chronic pain conditions in the development of ADRD. Given that ADRD is often underdiagnosed and progression is generally slow and insidious, and that the study follow-up period was short, under-ascertainment of ADRD is likely in this study, potentially biasing our risk estimates toward the null. In addition, given that we used a conservative method [75] for ascertaining chronic pain (i.e., ≥1 inpatient visit or two outpatient visits for any chronic pain conditions 90 days apart), and that NCPC is typically under-reported in medical claims data [76,77,78], NCPC may have been under-ascertained in this study, again potentially biasing risk estimates towards the null. Moreover, as sleep and mood disorders often accompany the development of ADRD, these disorders may have reflected prodromal ADRD in some who were diagnosed with incident ADRD. While we were able to adjust for a wide range of potential confounders, including smoking and BMI, we lacked information on certain lifestyle-related and other factors strongly linked to ADRD risk, including alcohol consumption, physical activity, genetic and familial predisposition, and social isolation [7,9]. Due to small cell sizes, we were also unable to adjust for Parkinson’s disease and related movement disorders, conditions which have been linked to chronic pain, as well as to mood and sleep disorders and cognitive impairment [79,80]. However, small cell sizes would also suggest that any residual confounding from these movement disorders is unlikely to explain our findings. Both ADRD and chronic pain have been associated with increased mortality [81,82,83], introducing potential survival bias. However, competing risk of death analyses yielded findings similar to those of our primary analyses, arguing against a substantive effect of survival bias on our study results. Finally, definitive conclusions regarding causality are not possible due to the short follow-up period in this study and the insidious nature of ADRD development and progression. However, while reverse causality cannot be ruled out, a growing literature suggests chronic pain can disrupt neurocognitive function and may increase risk for cognitive decline and incident dementia [18,65,84], whereas evidence for an inverse relationship remains sparse [22]. 

## 5. Conclusions

In this large, population-based study in a nationally representative sample of US community-dwelling elders enrolled in FFS Medicare, NCPC at baseline remained significantly and positively associated with risk for incident ADRD after adjustment for demographics, lifestyle factors, medical history, medications, and other factors. This association increased in magnitude with increasing NCPC burden and appeared to be partially mediated by the presence of mood and sleep disorders. Additional large, prospective studies with longer term follow-up are warranted to confirm our findings; to further elucidate the potentially important contribution of chronic pain to accelerated cognitive decline, new onset cognitive impairment and the development of ADRD; to clarify the potential mediating role of sleep and mood disorders; and to explore possible underlying mechanisms.

## Figures and Tables

**Table 1:** Baseline characteristics by non-cancer chronic pain conditions in older Medicare beneficiaries *, analyzed using linked data from the Medicare Current Beneficiary Survey and Medicare claims, 2001–2013.

Variables	Total		Any NCPC				*p*-Value ^§^
			Yes		No		
	N ^¥^	Wt.%	N	Wt.%	N	Wt. %	
***ALL*^¥^**	16,934	100	6369	100.0	10,565	100.0	
***Sociodemographics***							
**Sex**							<0.0001
Female	9669	56.9	4215	66.4	5454	51.5	
Male	7265	43.1	2154	33.6	5111	48.5	
**Age in Years**							<0.0001
65–69	4188	31.8	1335	26.4	2853	34.9	
70–74	3484	22.5	1230	22.1	2254	22.8	
75–79	3674	21.4	1411	22.8	2263	20.6	
80+	5588	24.3	2393	28.8	3195	21.7	
**Race/Ethnicity**							0.842
White	14,085	83.2	5293	83.4	8792	83.0	
Black	1159	6.7	438	6.7	721	6.7	
Hispanic	956	5.6	359	5.7	597	5.6	
Other	709	4.5	268	4.3	441	4.6	
**Education**							<0.0001
<High School	4605	25.0	1744	25.4	2861	24.7	
High School	6174	36.4	2349	37.0	3825	36.1	
Some College	2431	15.0	950	15.7	1481	14.6	
College	3675	23.6	1306	21.9	2369	24.6	
**Marital Status**							<0.0001
Married	9139	56.5	3208	52.8	5931	58.7	
Widowed	5803	30.6	2491	35.7	3312	27.6	
Divorce/separated	1455	9.5	490	8.3	965	10.2	
Other	532	3.3	179	3.1	353	3.5	
**Household Income**							<0.0001
<200% Federal Poverty Level	8260	45.9	3221	48.4	5039	44.6	
≥200% Federal Poverty Level	8674	54.1	3148	51.6	5526	55.4	
***Insurance***							
**Medicaid**							<0.0001
Yes	2147	11.9	1005	15.4	1142	9.9	
No	14,787	88.1	5364	84.6	9423	90.1	
**Private Insurance**							0.001
Yes	13,418	79.4	5147	80.8	8271	78.5	
No	3514	20.6	1221	19.2	2293	21.5	
***Residence***							
**Metropolitan Status**							<0.0001
Metro	11,375	70.6	4415	72.5	6960	69.5	
Rural	5558	29.4	1954	27.5	3604	30.5	
***Lifestyle Characteristics***							
**Body Mass Index**							<0.0001
Underweight	312	1.7	100	1.4	212	1.8	
Normal	5867	34.0	1984	30.4	3883	36.0	
Overweight	6709	40.0	2491	39.2	4218	40.5	
Obese	3881	24.3	1734	29.0	2147	21.7	
*Mean BMI 27.2 (±0.052)*							
**Smoking Status**							<0.0001
Current	1594	10.2	457	7.8	1137	11.5	
Past	7976	47.5	2965	47.2	5011	47.6	
Never	7326	42.4	2936	45.1	4390	40.8	
***Baseline Health History***							
***Physical Health Conditions***							
**Hypertension**							<0.0001
Yes	12,713	73.5	5263	82.0	7450	68.7	
No	4221	26.5	1106	18.0	3115	31.3	
**Heart disease**							<0.0001
Yes	4357	24.1	2097	31.5	2260	19.9	
No	12,577	75.9	4272	68.5	8305	80.1	
**Stroke**							<0.0001
Yes	2139	11.5	979	14.3	1160	9.9	
No	14,795	88.5	5390	85.7	9405	90.1	
**Diabetes**							<0.0001
Yes	3495	20.7	1468	23.5	2027	19.2	
No	13,439	79.3	4901	76.5	8538	80.8	
**Respiratory disease**							<0.0001
Yes	2423	14.2	1045	16.7	1378	12.7	
No	14,511	85.8	5324	83.3	9187	87.3	
**Cancer**							<0.0001
Yes	7101	40.3	2911	44.4	4190	38.0	
No	9833	59.7	3458	55.6	6375	62.0	
**Traumatic Brain Injury**							<0.0001
Yes	221	1.2	146	2.2	75	0.7	
No	16,713	98.8	6223	97.8	10,490	99.3	
**Rheumatoid Arthritis**							<0.0001
Yes	508	2.9	349	5.5	159	1.4	
No	16,426	97.1	6020	94.5	10,406	98.6	
**Lupus**							<0.0001
Yes	136	0.8	94	1.4	42	0.4	
No	16,798	99.2	6275	98.6	10,523	99.6	
**Number of NCPCs**							
0	6385	39.1	-	-	-	-	-
1	4194	24.4	-	-	-	-	-
2	3818	22.0	-	-	-	-	-
3	1908	10.9	-	-	-	-	-
≥4	629	3.7	-	-	-	-	-
***Sleep and Mood Disorders***							
**Sleep Disorder**							<0.0001
Yes	2728	15.8	1630	25.5	1098	10.2	
No	14,206	84.2	4739	74.5	9467	89.8	
**Depression**							<0.0001
Yes	1158	6.9	686	11.1	472	4.5	
No	15,776	93.1	5683	88.9	10,093	95.5	
**Anxiety**							<0.0001
Yes	862	5.1	492	7.9	370	3.5	
No	16,072	94.9	5877	92.1	10,195	96.5	
***Medication Use***							
**NSAIDs**							<0.0001
Yes	3241	18.6	1936	30.2	1305	12.0	
No	13,693	81.4	4433	69.8	9260	88.0	
**Opioid Analgesics**							<0.0001
Yes	3353	19.4	2059	32.2	1294	12.0	
No	13,581	80.6	4310	67.8	9271	88.0	
**Benzodiazepines**							<0.0001
Yes	1734	9.7	935	14.5	799	7.1	
No	15,200	90.3	5434	85.5	9766	92.9	
**Psychotropic medications**							<0.0001
Yes	2871	17.0	1448	23.3	1423	13.5	
No	14,063	83.0	4921	76.7	9142	86.5	

* Based on 11 pooled cohorts of 16,934 older community-dwelling adults (age > 65 years), enrolled in fee-for service Medicare, alive at the end of follow-up. ^¥^ Numbers for some variables may not add up to N = 16,934, due to missing values. ^§^ Statistically significant group differences in presence of NCPC were examined with Rao-Scott chi-square test. Abbreviation: NCPC, non-cancer chronic pain conditions; BMI, body mass index; NSAIDs, nonsteroidal anti-inflammatory drugs.

**Table 2:** Baseline characteristics by incident Alzheimer’s disease and related dementias (ADRD) in older Medicare beneficiaries *, analyzed using linked data from the Medicare Current Beneficiary Survey and Medicare claims, 2001-2013.

Variables	Incident ADRD				*p*-Value ^§^
	Yes		No		
	N	Wt. %	N	Wt. %	
***ALL*^¥^**	1149	6.8	15,785	93.2	
**Any NCPC**					<0.0001
Yes	798	6.6	9677	93.4	
No	351	4.3	6108	95.7	
**Number of NCPC’s**					<0.0001
0	351	4.3	6108	95.7	
1	304	6.2	3948	93.8	
2	273	6.0	3605	94.0	
3	155	7.3	1742	92.7	
≥4	66	17.3	382	82.7	
***Sociodemographic***					
**Sex**					<0.0001
Female	725	6.2	8944	93.8	
Male	424	5	6841	95	
**Age in Years**					<0.0001
65–69	101	1.9	4087	98.1	
70–74	139	4	3345	96	
75–79	220	5.9	3454	94.1	
80+	689	12	4899	88	
**Race/Ethnicity**					0.066
White	922	5.5	13,163	94.5	
Black	106	7.4	1053	92.6	
Hispanic	70	5.9	886	94.1	
Other	51	6.5	658	93.5	
**Education**					<0.0001
<High School	417	7.8	4188	92.2	
High School	398	5.5	5776	94.5	
Some College	141	4.8	2290	95.2	
College	186	4.1	3489	95.9	
**Marital Status**					<0.0001
Married	494	4.5	8645	95.5	
Widowed	541	8.2	5262	91.8	
Divorce/separated	76	4.4	1379	95.6	
Other	38	6	494	94	
**Household Income**					<0.0001
<200% Federal Poverty Level	699	7.4	7561	92.6	
≥200% Federal Poverty Level	450	4.3	8224	95.7	
***Insurance***					
**Medicaid**					<0.0001
Yes	223	9.1	1924	90.9	
No	926	5.2	13,861	94.8	
**Private Insurance**					<0.0001
Yes	847	5.3	12,571	94.7	
No	300	7.2	3214	92.8	
***Residence***					0.649
**Metropolitan Status**					
Metro	787	5.7	10,588	94.3	
Rural	362	5.5	5196	94.5	
***Lifestyle Characteristics***					
**Body Mass Index**					<0.0001
Underweight	43	12.2	269	87.8	
Normal	484	6.9	5383	93.1	
Overweight	417	5.2	6292	94.8	
Obese	195	4.3	3686	95.7	
**Smoking Status**					<0.0001
Current	96	4.8	1498	95.2	
Past	472	5	7504	95	
Never	580	6.6	6746	93.4	
***Baseline Health History***					
***Physical Health Conditions***					
**Hypertension**					<0.0001
Yes	935	6.3	11,778	93.7	
No	214	4.1	4007	95.9	
**Heart disease**					<0.0001
Yes	368	7.6	3989	92.4	
No	781	5.1	11,796	94.9	
**Stroke**					<0.0001
Yes	281	11.9	1858	88.1	
No	868	4.9	13,927	95.1	
**Diabetes**					0.032
Yes	264	6.4	3231	93.6	
No	885	5.5	12,554	94.5	
**Respiratory disease**					0.576
Yes	170	5.9	2253	94.1	
No	979	5.6	13,532	94.4	
**Cancer**					0.043
Yes	505	6.2	6596	93.8	
No	644	5.3	9189	94.7	
**Traumatic Brain Injury**					<0.0001
Yes	30	12.8	191	87.2	
No	1119	5.6	15,594	94.4	
**Rheumatoid Arthritis**					0.247
Yes	39	6.8	469	93.2	
No	1110	5.6	15,316	94.4	
**Lupus**					0.119
Yes	13	8.9	123	91.1	
No	1136	5.7	15,662	94.3	
***Sleep and Mood Disorders***					
**Sleep Disorder**					<0.0001
Yes	267	8.5	2461	91.5	
No	882	5.2	13,324	94.8	
**Depression**					<0.0001
Yes	170	12.9	988	87.1	
No	979	5.1	14,797	94.9	
**Anxiety**					<0.0001
Yes	109	10.9	753	89.1	
No	1040	5.4	15,032	94.6	
***Medication Use***					
**NSAIDs**					0.496
Yes	226	6	3015	94	
No	923	5.6	12,770	94.4	
**Opioid Analgesics**					<0.0001
Yes	266	6.9	3087	93.1	
No	883	5.4	12,698	94.6	
**Benzodiazepines**					<0.0001
Yes	167	8.5	1567	91.5	
No	982	5.4	14,218	94.6	

* Based on 11 pooled cohorts of 16,934 older community-dwelling adults (age ≥ 65 years), enrolled in fee-for service Medicare, alive at the end of follow-up. ^¥^ Numbers for some variables may not add up to N = 1149, due to missing values. ^§^ Statistically significant group differences in presence of NCPC were examined with Rao-Scott chi-square test. Abbreviations: NCPC, non-cancer chronic pain conditions; ADRD, Alzheimer’s disease and related dementias; NSAIDs, nonsteroidal anti-inflammatory drugs.

**Table 3:** Association of baseline non-cancer chronic pain conditions (NCPC) and burden to incident Alzheimer’s disease and related dementias (ADRD) in older Medicare beneficiaries *: analysis using linked Medicare Current Beneficiary Survey and Medicare claims data, 2001–2013 (odds ratios (OR) and adjusted odds ratios (AOR) with 95% confidence intervals (CI) calculated using separate logistic regression models).

NCPC Presence/Number	Unadjusted Model		Model 1 ^¥^		Model 2 ^¥¥^		Model 3 ^ⱡⱡ^	
	OR (95% CI)	*p*	AOR (95% CI)	*p*	AOR (95% CI)	*p*	AOR (95% CI)	*p*
Any NCPC ** vs. None	1.54 (1.34,1.78)	<0.0001	1.33 (1.14,1.53)	<0.0001	1.28 (1.10,1.48)	0.001	1.25 (1.08,1.45)	0.003
**Number of NCPCs**								
None (referent)	1.00		1.00		1.00		1.00	
One	1.47 (1.26,1.71)	<0.0001	1.27 (1.08,1.50)	0.0039	1.24 (1.05,1.46)	0.0125	1.23 (1.04,1.45)	0.0179
Two	1.42 (1.20,1.68)	<0.0001	1.25 (1.05,1.49)	0.0121	1.22 (1.02,1.45)	0.0278	1.20 (1.01,1.43)	0.0435
Three	1.75 (1.42,2.16)	<0.0001	1.45 (1.17,1.80)	0.0007	1.38 (1.11,1.71)	0.0041	1.34 (1.07,1.68)	0.0122
Four or more	3.03 (2.14,4.29)	<0.0001	2.32 (1.62,3.31)	<0.0001	1.98 (1.36,2.89)	0.0004	1.91 (1.31,2.80)	0.0008
*** P for linear trend ^++^***		<0.0001		<0.0001		<0.0001		<0.0001
**Number of NCPCs**								
(per additional NCPC)	1.23 (1.16,1.30)	<0.0001	1.16 (1.09,1.23)	<0.0001	1.13 (1.06,1.20)	0.0002	1.12 (1.05,1.20)	0.0007

* Based on 11 pooled cohorts of 16,934 older community-dwelling adults (age > 65 years), enrolled in fee-for-service Medicare. ** Including back and neck pain, headache and migraine, joint pain, neuropathic pain and osteoarthritis. ^¥^ Socio-demographics: sex, age, race/ethnicity, education, income, Medicaid insurance, private insurance, marital status, region. ^¥¥^ Lifestyle and chronic physical health conditions: smoking, body mass index, hypertension, diabetes, heart disease, cancer, respiratory disorder, history of stroke, traumatic brain injury. ^ⱡⱡ^ Analgesics: nonsteroidal anti-inflammatory drugs and opioid analgesics. ^++^ Regression results from polynomial contrast for linear relation indicate a strong linear effect of NCPC on risk of ADRD.

**Table 4:** Association of baseline non-cancer chronic pain conditions to incident Alzheimer’s disease and related dementias in older Medicare beneficiaries *: competing risk analysis using linked Medicare Current Beneficiary Survey (MCBS) and Medicare claims data, 2001–2013 (odds ratios (OR) and adjusted odds ratios (AOR) with 95% confidence intervals (CI) calculated from multinomial logistic regression).

Any Non-Cancer Chronic Pain Condition (NCPC) **	Alive at Follow-Up, No ADRD (Referent)		Alive at Follow-Up, Incident ADRD		Died before Follow-Up, Incident ADRD		Died before Follow-Up, No ADRD	
	OR	*p*	OR (95% CI)	*p*	OR (95% CI)	*p*	OR (95% CI)	*p*
**Model 1**. Unadjusted model	1.00	-	1.55 (1.34,1.78)	<0.0001	1.29 (1.03,1.62)	0.029	1.25 (1.12,1.40)	<0.0001
**Model 2**. Adjusted for sociodemographics ^¥^	1.00	-	1.34 (1.16,1.55)	<0.0001	1.10 (0.87,1.40)	0.42	1.21 (1.07,1.36)	0.0024
**Model 3**. Also adjusted for lifestyle and chronic physical health conditions ^¥¥^	1.00	-	1.28 (1.11,1.49)	0.0009	1.03 (0.79,1.34)	0.8287	1.05 (0.93,1.18)	0.4461
**Model 4**. Also adjusted for NSAID and opioid analgesic use	1.00	-	1.26 (1.08,1.46)	0.0026	0.99 (0.75,1.31)	0.96	0.97 (0.85,1.10)	0.60

* Based on 11 pooled cohorts of older community-dwelling adults (age > 65 years), enrolled in fee-for-service Medicare. ** Referent for Any NCPC ‘No NCPC’. ^¥^ Sex, age, race/ethnicity, education, income, Medicaid insurance, private insurance, marital status, region. ^¥¥^ Smoking status, body mass index, hypertension, diabetes, heart disease, cancer, respiratory illnesses, history of stroke and traumatic brain injury.

**Table 5:** Association of baseline non-cancer chronic pain conditions (NCPC’s) and burden to incident Alzheimer’s disease and related dementias in older Medicare beneficiaries *: analysis using linked Medicare Current Beneficiary Survey and Medicare claims data, 2001–2013: Potential mediating influence of diagnosed mood and sleep disorders (odds ratios (OR) and adjusted odds ratios (AOR) with 95% confidence intervals (CI)).

NCPC Presence/Number	Fully Adjusted Model ^¥^		Model ^¥^ + Mood Disorders ^§^		Model ^¥^ + Sleep Disorders ^§§^		Model ^¥^ + Mood and Sleep Disorders	
	OR (95% CI)	*p*	OR (95% CI)	*p*	OR (95% CI)	*p*	OR (95% CI)	*p*
Any NCPC vs. None **	1.25 (1.08,1.45)	0.0032	1.17 (1.01,1.37)	0.0388	1.20 (1.04,1.39)	0.0155	1.18 (1.01,1.38)	0.0336
**Number of NCPCs**								
None (referent)	1.00 (referent)		1.00 (referent)		1.00 (referent)		1.00 (referent)	
One	1.23 (1.04,1.45)	0.0179	1.19 (1.01,1.41)	0.0408	1.20 (1.02,1.43)	0.0311	1.17 (0.99,1.39)	0.0594
Two	1.20 (1.01,1.43)	0.0435	1.13 (0.95,1.36)	0.1722	1.16 (0.97,1.38)	0.1008	1.11 (0.92,1.32)	0.2713
Three	1.34 (1.07,1.68)	0.0122	1.22 (0.96,1.55)	0.0962	1.25 (0.99,1.58)	0.0589	1.17 (0.92,1.48)	0.2112
Four or more	1.91 (1.31,2.80)	0.0008	1.57 (1.06,2.34)	0.0255	1.66 (1.12,2.45)	0.0113	1.41 (0.94,2.12)	0.0941
***p for linear trend*^++^**		<0.0001		<0.0001		<0.0001		<0.0001
**Number of NCPCs**								
(per additional NCPC)	1.12 (1.05,1.20)	0.0007	1.08 (1.01,1.16)	0.027	1.09 (1.02,1.16)	0.0101	1.06 (0.99,1.13)	0.1001

* Based on 11 pooled cohorts of older community-dwelling adults (age > 65 years), enrolled in fee-for-service Medicare. ** Including back or neck pain, headache and migraine, joint pain, neuropathic pain, and osteoarthritis. ^¥^ sex, age, race/ethnicity, education, income, Medicaid insurance, private insurance, marital status, region, smoking status, body mass index, chronic physical health conditions (hypertension, diabetes, heart disease, cancer, respiratory disorder, history of stroke, traumatic brain injury), NSAID and opioid analgesics. ^§^ Depression and anxiety; ^§§^ insomnia-related sleep disorders. ^++^ Regression results from polynomial contrast for linear relation indicate a strong linear effect of NCPC on risk of ADRD.

## Supplementary Materials

The following are available online at https://www.mdpi.com/1660-4601/17/15/5454/s1, Figure S1: ADRD-NCPC Study Cohort: Medicare Current Beneficiary Survey (MCBS), 2001–2013, Table S1: ICD-9_Clinial Modification (ICD-9-CM) Diagnostic Codes.
