# Supplementary material for: Non-Cancer Chronic Pain Conditions and Risk for Incident Alzheimer’s Disease and Related Dementias in Community-Dwelling Older Adults: A Population-Based Retrospective Cohort Study of United States Medicare Beneficiaries, 2001–2013
Source: Int J Environ Res Public Health. 2020 Jul 29;17(15):5454. doi: 10.3390/ijerph17155454 (PMC7432104; doi:10.3390/ijerph17155454)
Supplement: Supplementary file 1 [file ijerph-17-05454-s001.zip › S1Figure-23Jun2020-IJERPH.docx]

# Supplementary 1: Figure (S1 Figure)

# ADRD-NCPC Study Cohort: Medicare Current Beneficiary Survey (MCBC), 2001 - 2013

# (Stepwise exclusions, based on the baseline characteristics drawn from the baseline year of each of the 11 MCBS study cohorts)

**ALL (N = 45535)**

N = 4471 (2001-2003) N = 4308 (2002_2004)

N = 4337 (2003_2005) N = 4166 (2004_2006)

N = 4192 (2005_2007) N = 4242 (2006_2008)

N = 4340 (2007_2009) N = 4026 (2008_2010)

N = 3273 (2009_2011) N = 3992 (2010_2012)

N = 4188 (2011_2013)

**Older Adults (age > 65 years) (N = 37852)**

N = 3728 (2001-2003) N = 3624 (2002_2004)

N = 3607 (2003_2005) N = 3484 (2004_2006)

N = 3465 (2005_2007) N = 3511 (2006_2008)

N = 3608 (2007_2009) N = 3351 (2008_2010)

N = 2714 (2009_2011) N = 3333 (2010_2012)

N = 3427 (2011_2013)

Reason for exclusion:

- Under Age 65 at baseline (N=7683)

**Older Adults (age > 65 years), Community Dwelling (N = 34441)**

N = 3381 (2001-2003) N = 3265 (2002_2004)

N = 3310 (2003_2005) N = 3147 (2004_2006)

N = 3137 (2005_2007) N = 3197 (2006_2008)

N = 3282 (2007_2009) N = 3066 (2008_2010)

N = 2486 (2009_2011) N = 3002 (2010_2012)

N = 3168 (2011_2013)

Reasons for exclusion:

- Not community-dwelling during the baseline year (N=3411)

**Older Adults (age > 65 years), Community Dwelling, FFS (N = 23445)**

N = 2468 (2001-2003) N = 2440 (2002_2004)

N = 2531 (2003_2005) N = 2413 (2004_2006)

N = 2392 (2005_2007) N = 2278 (2006_2008)

N = 2067 (2007_2009) N = 1792 (2008_2010)

N = 1454 (2009_2011) N = 1646 (2010_2012)

N = 1964 (2011_2013)

Reasons for exclusion:

- Not enrolled in fee for service at baseline (N=10996)

**Older Adults (age > 65 years), Community Dwelling, FFS, Full-year enrolled (N = 22525)**

N = 2382 (2001-2003) N = 2357 (2002_2004)

N = 2433 (2003_2005) N = 2318 (2004_2006)

N = 2303 (2005_2007) N = 2187 (2006_2008)

N = 1976 (2007_2009) N = 1716 (2008_2010)

N = 1401 (2009_2011) N = 1567 (2010_2012)

N = 1885 (2011_2013)

Reasons for exclusion

- Not enrolled for full year during the baseline year (N=920)

**Older Adults (age > 65 years), Community Dwelling, FFS, Full-year enrolled, Alive (N = 17812)**

N = 1957 (2001-2003) N = 1868 (2002_2004)

N = 1967 (2003_2005) N = 1871 (2004_2006)

N = 1838 (2005_2007) N = 1734 (2006_2008)

N = 1556 (2007_2009) N = 1343 (2008_2010)

N = 1104 (2009_2011) N = 1209 (2010_2012)

N = 1365 (2011_2013)

Reasons for exclusion:

- Not alive/missing during baseline and follow-up years (N=4713)

Reasons for exclusion:

- Not ADRD-free during baseline year (N= 878)

**Final Study Sample**

**Older Adults (age > 65 years), Community Dwelling, FFS, Full-year enrolled, Alive, ADRD-free (N = 16934)**

N = 1874 (2001-2003) N = 1768 (2002_2004)

N = 1884 (2003_2005) N = 1766 (2004_2006)

N = 1735 (2005_2007) N = 1650 (2006_2008)

N = 1495 (2007_2009) N = 1278 (2008_2010)

N = 1034 (2009_2011) N = 1159 (2010_2012)

N = 1291 (2011_2013)
