# Supplementary material for: Non-Cancer Chronic Pain Conditions and Risk for Incident Alzheimer’s Disease and Related Dementias in Community-Dwelling Older Adults: A Population-Based Retrospective Cohort Study of United States Medicare Beneficiaries, 2001–2013
Source: Int J Environ Res Public Health. 2020 Jul 29;17(15):5454. doi: 10.3390/ijerph17155454 (PMC7432104; doi:10.3390/ijerph17155454)
Supplement: Supplementary file 1 [file ijerph-17-05454-s001.zip › S1Table-23Jun2020-IJERPH.docx]

| Conditions | ICD-9-CM codes |
| --- | --- |
| Depressive disorders | 296.20, 296.21, 296.22, 296.23, 296.24, 296.25, 296.26, 296.30, 296.31, 296.32, 296.33, 296.34, 296.35, 296.36, 300.4, 311, V79.0 |
| Anxiety disorders | 293.84, 300.00, 300.01, 300.02, 300.09, 300.10, 300.20, 300.21, 300.22, 300.23, 300.29, 300.3, 300.5, 300.89, 300.9, 308.0, 308.1, 308.2, 308.3, 308.4, 308.9, 309.81, 313.0, 313.1, 313.21, 313.22, 313.3, 313.82, 313.83 |
| Sleep disorders | 342 -347, 468, 784, 3273, 3274, 3278, 4118, 5301, 7872, 27803, 29182, 29285, 30741, 30744, 30746, 30747, 30749, 32711, 32713, 32714, 32715, 32721, 32722, 32723, 32724, 32725, 32726, 32727, 32729, 32731, 32732, 32733, 32734, 32735, 32736, 32741, 32742, 32743, 32744, 32749, 32751, 32752, 32753, 32759, 33394, 34701, 36816, 77081, 77082, 78604, 78836, 32723, 30741, 30749, 32730, 32731, 32732, 32733, 32735, 32736 |
| Alzheimer’s disease and related dementias | 290.0-290.3, 331.0-331.2, 331.7, 331.8 |
| Non-cancer chronic pain conditions (CMS CCW codes using CCS software) | 203 (osteoarthritis); 201, 204, 211, 212, 54 and 225 (Joint pain); 205, 209, 217, 231, 232 (back and neck pain); 84, 670 (head and migraine pain); 95 (neuropathic pain). |
| Traumatic brain injury | 800-804, 850-854, 950, 959, 995 |
| Ischemic heart diseases | 410.00, 410.01, 410.02, 410.10, 410.11, 410.12, 410.20, 410.21, 410.22, 410.30, 410.31, 410.32, 410.40, 410.41, 410.42, 410.50, 410.51, 410.52, 410.60, 410.61, 410.62, 410.70, 410.71, 410.72, 410.80, 410.81, 410.82, 410.90, 410.91, 410.92, 411.0, 411.1, 411.81, 411.89, 412, 413.0, 413.1, 413.9, 414.00, 414.01, 414.02, 414.03, 414.04, 414.05, 414.06, 414.07, 414.12, 414.2, 414.3, 414.4, 414.8, 414.9, 410.01, 410.11, 410.21, 410.31, 410.41, 410.51, 410.61, 410.71, 410.81, 410.91 |
| Diabetes Mellitus | 249.00, 249.01, 249.10, 249.11, 249.20, 249.21, 249.30, 249.31, 249.40, 249.41, 249.50, 249.51, 249.60, 249.61, 249.70, 249.71, 249.80, 249.81, 249.90, 249.91, 250.00, 250.01, 250.02, 250.03, 250.10, 250.11, 250.12, 250.13, 250.20, 250.21, 250.22, 250.23, 250.30, 250.31, 250.32, 250.33, 250.40, 250.41, 250.42, 250.43, 250.50, 250.51, 250.52, 250.53, 250.60, 250.61, 250.62, 250.63, 250.70, 250.71, 250.72, 250.73, 250.80, 250.81, 250.82, 250.83, 250.90, 250.91, 250.92, 250.93, 357.2, 362.01, 362.02, 362.03, 362.04, 362.05, 362.06, 366.41 |
| Stroke | 430, 431, 433.01, 433.11, 433.21, 433.31, 433.81, 433.91, 434.00, 434.01, 434.10, 434.11, 434.90, 434.91, 435.0, 435.1, 435.3, 435.8, 435.9, 436, 997.02. Excluding TBI codes: 800-804; 850-854 |
| Hypertension | 362.11, 401.0, 401.1, 401.9, 402.00, 402.01, 402.10, 402.11, 402.90, 402.91, 403.00, 403.01, 403.10, 403.11, 403.90, 403.91, 404.00, 404.01, 404.02, 404.03, 404.10, 404.11, 404.12, 404.13, 404.90, 404.91, 404.92, 404.93, 405.01, 405.09, 405.11, 405.19, 405.91, 405.99, 437.2. |
| Respiratory illnesses | 493.00, 493.01, 493.02, 493.10, 493.11, 493.12, 493.20, 493.21, 493.22, 493.81, 493.82, 493.90, 493.91, 493.92, 490, 491.0, 491.1, 491.8, 491.9, 492.0, 492.8, 491.20, 491.21, 491.22, 494.0, 494.1, 49 |
